# Inflammatory Myofibroblastic Tumor of Genitourinary Tract Beyond Collecting System: A Rare Case Report With Literature Review

**DOI:** 10.1097/MD.0000000000001706

**Published:** 2015-10-23

**Authors:** Ling-Fan Xu, Jun Zhou, Chao-Zhao Liang

**Affiliations:** From the Department of Urology, The First Affiliated Hospital of Anhui Medical University, Hefei, China (LFX, JZ, C-ZL).

## Abstract

Inflammatory myofibroblastic tumor (IMT) rarely arises in genitourinary tract especially beyond collecting system, which determines the unspecific clinic symptoms and sometimes can mimic malignancy. Therefore, IMT's diagnosis may usually be a pitfall. This case report characterizes a 35-year-old woman with a history of lower quadrant lasting pain followed by fever. Furthermore, radiologic examinations revealed that there were 2 lesions located in left adrenal area and left renalis. Owing to the anatomic complexity, the surgical resection was not complete. The pathologic diagnosis of the lesions was IMT. Adjuvant nonsteroids anti-inflammatory drugs were administrated after the operation. The symptoms were controlled finally and no further growing lesion was observed during a 1-year follow-up.

Inflammatory myofibroblastic tumor is rare in genitourinary tract beyond the collecting system. Diagnosis should be based on histopathology. Presently, the authors report this rare case with the aim to share the experience regarding differential diagnosis and therapy.

## INTRODUCTION

Inflammatory myofibroblastic tumor (IMT) is a rare mesenchymal tumor of unknown etiology and pathogenesis composed of neoplastic myofibroblasts with inflammatory infiltrate, which can arise in multiple anatomic locations. Therefore, the patients often exhibit highly variable and unpredictable clinical behaviors. For adults, IMT mainly initiates in respiratory and alimentary systems, such as lung, liver, colon, spleen, pancreas, etc.^1–3^ By contrast, IMT seldom occurs in the genitourinary tract and most of the ones that have been reported are urinary bladder and prostate lesions.^4^

Herein, we present a special case of a 35-year-old woman with adrenal area and renalis IMT, which, to the best of our knowledge, have hardly been reported in other medical institutions.

## CASE REPORT

A 35-year-old woman was initially admitted to our department on June 15, 2014 (The First Affiliated Hospital of Anhui Medical University) with a 1-month history of lasting pain in left lower quadrant of abdomen, which exacerbated at night. Furthermore, case history was noncontributory, such as gross hematuria, dysuria, nausea, vomit, etc.

**FIGURE 1:**
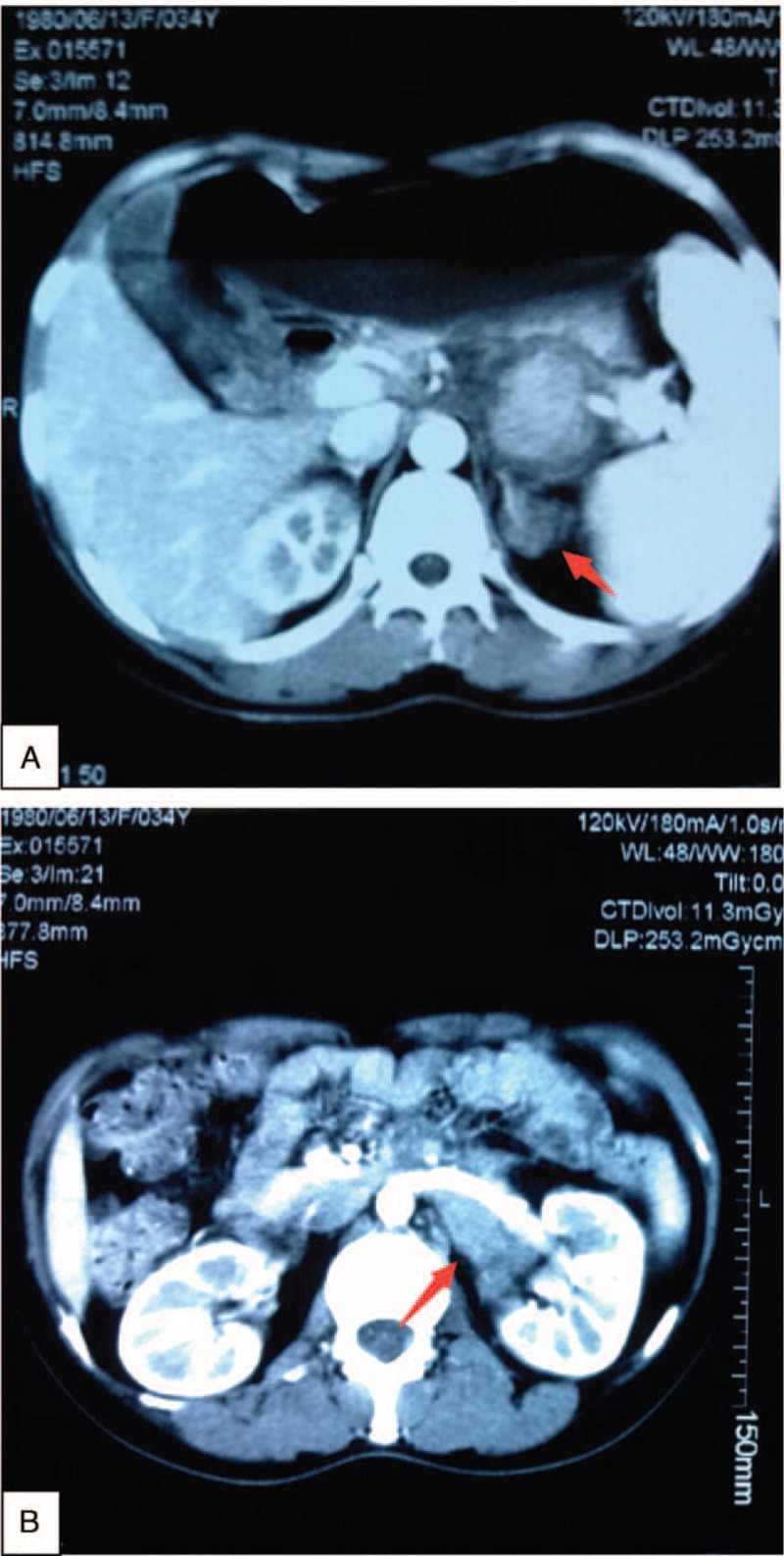
Contrast-enhanced computed tomography of abdomen reveals 2 conspicuous unclear mass located in the left adrenal area and left renalis of approximately 4.5 cm × 3 cm and 3 cm × 2 cm, respectively (A-B).

Contrast-enhanced computed tomography of abdomen revealed 2 conspicuous unclear border masses located in the left adrenal area and left renalis of approximately 4.5 cm × 3 cm and 3 cm × 2 cm, respectively (Fig. 1A-B).

After admission, the patient's body temperature became ascending and the highest was 39.8 °C. We, however, could not find any relevant infectious factors. The whole blood culture results were negative. Considering the symptoms and radiologic presentations, the first clinical impression was malignant lymphoma.

On June 17, we undertook a surgical resection. Because the renalis mass adjacent to the renal artery, which could not be separated, we excised the whole mass in left adrenal area meanwhile resected the renalis mass as much as possible to make clear the pathologic character of the lesions.

**FIGURE 2:**
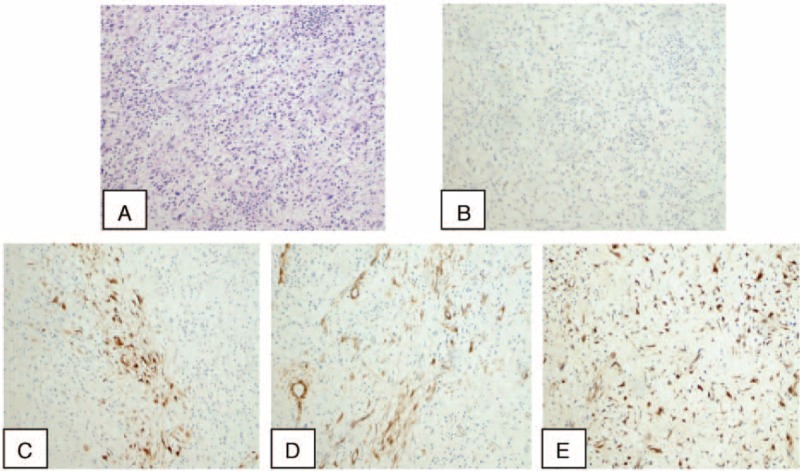
A, The histopathologic examination demonstrates an abnormal proliferation of spindle cells in a fascicular and myxoid pattern with no significant nuclear atypia accompanied by inflammatory cells (hematoxylin and eosin stain, original magnification, ×100). Immunohistochemical appearance shows that calponin, anaplastic lymphoma kinase, smooth muscle actin, and vimentin are positive in inflammatory myofibroblastic tumor of this case (B-E ×100).

The histopathologic examination of the masses demonstrated an abnormal proliferation of spindle cells in a fascicular and myxoid pattern with no significant nuclear atypia accompanied by inflammatory cells, including lymphocytes, eosinophils, and plasma cells. The immunohistochemical stains illustrated that spindle cells were positive for anaplastic lymphoma kinase (ALK), smooth muscle actin (SMA), calponin, and vimentin whereas negative for CD15, CD23, CD30, CD34, creatinine kinase, or leukocyte common antigen (Fig. 2A-E).

In postoperative period, we treated the patient with adjuvant nonsteroids anti-inflammatory drugs (NSAIDs). Following the diclofenac sodium and lidocaine hydrochloride injection therapy (95 mg/d, administered intramuscular injection for the first 3 days), the patient's clinical symptoms improved. She was subsequently kept on the oral substitution for another week (50 mg of diclofenac sodium enteric-coated tablets per day). The body temperature gradually turned normal and the patient recovered uneventfully. Frequent re-examinations were required. During the most recent follow-up in March 2015, the woman remained healthy and fit without any uncomfortable complains and we could not find any neonatal lesions from the radiologic examination compared with the former one.

## DISCUSSION

Inflammatory myofibroblastic tumor is an unusual spindle cell proliferative lesion, which was first reported by Brunn et al^5^ on 2 cases of “myoma of the lung” in 1939, and thus, in general, it is classified under the pulmonary and extrapulmonary sites category.^6^ During the last 50 years, IMT has been attributed to numerous nomenclature, such as inflammatory pseudotumor, pseudosarcomatous myofibroblastic, fibromyxoid lesion, and plasma cell granuloma.^4^ Inflammatory myofibroblastic tumor, however, has been identified because its histopathologic features have been revealed gradually. Although the exact pathogenesis remains unknown, all these lesions share the key pathologic differentiation: a variety of inflammatory cells, plasma cells, and/or lymphocytes accompany with a dominant spindle cell proliferation, which have been demonstrated as myofibroblasts, according to the World Health Organization in 1994.^7^

The histologic presentation of IMT may range broadly from tumors at different anatomic sites to those at the same site. Three typical appearances have been described: myofibroblasts surrounded by a stellate or spindled appearance, which are mainly found in the genitourinary; more densely packed stromal cells interspersed with an inflammatory component, which may show focal nodular lymphoid hyperplasia; and hypocellular stroma.^4^ These 3 patterns can either mix in 1 tumor or exhibit independently. In a previous systematic review by Teoh et al,^8^ the myxoid or vascular pattern took up a considerable proportion, with the figure standing at 83.8%, of the total number of all the IMT patents. The fibrous hypocellular pattern occupied the smallest percentage, which accounted 5.9%. And the compact spindle cell pattern contributed 55.9%. Approximately, 41.2% of the total patients had mixed histologic patterns. Intermingling bundles of fibrocollagenous tissue and inflammatory cells were shown in our case, which supported the IMT diagnosis.

Although the morbidity of IMT has a wide spectrum of ages (between 7 days and 88 years) in both sexes, it is more frequently seen in youths and has a sex predilection for female.^9^ Studies have shown that it is rare to find IMT in genitourinary tract, let alone kidney, urethra, ureter, and prostate. Furthermore, among those cases, most arise in urinary bladder.^10^ The most common symptom is painless hematuria either with or without clots. Others include abdominal or pelvic pain, dysuria, and infection.^10^ Systemic symptoms, such as fever and weight loss are seldom observed in clinic. Owing to the lacking of specification of clinical symptoms and radiologic examinations, the clinical diagnosis of IMT is quite difficult. In this case, the primary symptom of the patient was fever, rather than any urinary system discomfort. The temperature type is intermittent fever. In addition, the contrast-enhanced computed tomography examination indicated 2 unclear border masses located in adrenal area and renalis, respectively. These features were very similar to the performance of malignant lymphoma cases that we had observed before. Therefore, malignant lymphoma emerged as the first clinic impression.

As we know, all the tumor's attributes should be diagnosed by histologic examination, certainly, including IMT. Unfortunately, the differential is not distinct because the overlapping of inflammatory pseudotumor, pseudosarcomatous myofibroblastic, or fibromyxoid lesion, plasma cell granuloma, especially the postoperative spindle cell nodule, which runs a benign reactive myofibroblastic proliferation of the genitourinary tract that arises exclusively after instrumentation, usually a surgical procedure such as transurethral resection of bladder tumor.^11^ In the recent 15 years, the consequence of ALK expressed in IMT has made it a relative distinct entity when it comes to immunohistochemical and molecular characteristics.^8^ Anaplastic lymphoma kinase rearrangements were first reported in the anaplastic large cell lymphoma, and Cessna et al^12^ described that more than 40% IMT patients had the abnormalities of chromosome 2p23 with expression of ALK and p80. These genetic bases demonstrate that IMT is neoplastic rather than simply reactive in nature. It also makes ALK a hallmark for IMT histologic diagnosis. Besides, previous literature have demonstrated that the spindle cells of IMT reacted immunohistochemically with vimentin, calponin, and SMA but were usually negative for desmin.^13^ Qiu et al^14^ reported that among the 18 IMT patients, the proportion of expression of calponin and SMA were 100 and 94%, respectively, whereas desmin expressed less frequently (7/21, 33%). Similarly, in a case of a 9-month-old boy with mesenteric IMT, Buccoliero et al^15^ found that the tumor cells were positive for vimentin whereas negative for CD34 and S100 protein. Generally, the lesions are negative for CD15, CD34, CD117 (c-kit), etc.^16^ As illustrated in both the current series and others, it, however, is not always possible to identify SMA, calponin, and vimentin positivity or positivity for other markers of myofibroblastic differentiation. Many factors (eg, age, anatomic site, etc.) may result in different expressions.^17^ In our case, the spindle cells were positive for ALK, vimentin, calponin, and SMA, all of which confirmed the IMT diagnosis in histologic aspect and it was considered as a mixed pattern from its histologic features.

Owing to the fact that IMT has been confirmed in a spectrum of anatomic locations, there are various treatment modalities, including surgery, steroids, NSAIDs, radiotherapy, and chemotherapy. Of these treatments, surgical resection remains the priority for all the IMT lesions if not prohibited by anatomic location. The role of chemotherapy and radiation therapy is unclear upon the limited experience. Dishop et al^18^ reported a pediatric IMT patient received chemotherapy, and was tumor free in the 2 years following the incomplete surgical resections. Radiation therapy has been suggested to be effective to a certain degree in pulmonary IMT, according to Imperato et al.^19^ If complete resection is not possible, the alternatives of chemotherapy combined with radiation therapy, typically biopsy or observation could be taken into consideration. Steroids and NSAIDs may be regarded as an adjuvant approach to reducing the surrounding inflammation. There are several case reports of IMT being successfully treated with NSAIDs.^20–22^ According to Applebaum et al,^23^ because IMTs are composed of organized vasculature and inflammatory tissue admixed with myfibroblasts, and the identification of a substantial presence of vascular endothelial growth factor (VEGF) and cyclooxygenase 2 (Cox-2) enzyme in IMTs, they hypothesized that NSAIDs can be applied to these masses to disrupt the angiogenesis, specifically by interfering with VEGF signaling via Cox-2 inhibition. In their study, all the samples are positive of VEGF and Cox-2 immunostaining, supporting the idea that interference with Cox-2 angiogenic pathway is a possible strategy. The diclofenac sodium and lidocaine hydrochloride injection, consisted of diclofenac sodium (75 mg) and lidocaine hydrochloride (20 mg), is a Cox-2 inhibitor that has been widely used in clinic to reduce the inflammation. In our case, because of the anatomic complexity, a complete resection of the 2 masses was not possible. In the postoperative stage, we treated the patient with the diclofenac sodium and lidocaine hydrochloride injection as part of the adjuvant therapy. It finally came with an exciting result. The symptom disappeared and the patient was back to a normal life. A close follow-up was undergoing and the rest of the mass was not growing.

Although IMT rarely processes metastasis, Coffin et al^24^ demonstrated that the recurrence happened in approximately 25% of IMT patients and particularly when the lesions were extrapulmonary. In addition, because IMT has been reported to exhibit aggressive local behavior and locally destructive variants, a close follow-up is highly recommended, especially for the patients whose lesions cannot be operatively extirpated completely.

## CONCLUSIONS

From the current case, we demonstrate that although IMT is rare in genitourinary tract, it can still be found even in renalis and adrenal area. Diagnosis of IMT can be misled to other system diseases such as malignant lymphoma because of its atypical appearances. A definitive diagnosis should depend on histopathology. Complete excision should be considered firstly for localized lesion as long as the functional and anatomic conditions are acceptable. Whereas, on condition that the surgical resection is not satisfactory, steroids and NSAIDs may be used to control the surrounding inflammation to achieve the maximum clinical efficacy. Meanwhile, a long-term follow-up and close surveillance are indispensable according to its histologic resemblance to certain malignant tumors.

### Ethics Statement

Informed consents were signed by this patient and the investigation was evaluated thoroughly and then approved strictly according to the regulations by the Ethics Committees on Human Research of The First Affiliated Hospital of Anhui Medical University (Approval No. PJ20150808).
